# Comparison of perioperative stress in patients undergoing thyroid surgery with and without neuromonitoring—a pilot study

**DOI:** 10.1007/s00423-016-1457-5

**Published:** 2016-06-14

**Authors:** Dominika Babińska, Marcin Barczyński, Tomasz Osęka, Maciej Śledziński, Andrzej J. Łachiński

**Affiliations:** 1 Department of General, Endocrinological and Transplantation Surgery, Medical University of Gdańsk, 17 Mariana Smoluchowskiego Street, 80-214 Gdańsk, Poland; 2 Department of Endocrine Surgery, 3rd Chair of General Surgery, Jagiellonian University Medical College, Kraków, Poland; 3 Department of General Surgery, Self-Dependent Health Care Unit of the Ministry of Interior in Gdańsk, Gdańsk, Poland

**Keywords:** Intraoperative neuromonitoring, Thyroidectomy, Stress, Anxiety

## Abstract

**Purpose:**

A comprehensive psychological comparison of preoperative stress in patients scheduled for thyroidectomy with versus without intraoperative neurophysiologic monitoring (IONM) has never been reported. The aim of this study was to assess whether a planned utilization of IONM had any effect on the reduction of stress and anxiety level before and after thyroid surgery.

**Methods:**

The outcomes of 32 patients scheduled for thyroidectomy with IONM were compared to the outcomes of a carefully matched control group of 39 patients operated on without IONM. All the patients were tested before the surgery and at 1–7 days postoperatively employing psychological self-report instruments: the Depression Anxiety Stress Scales (DASS), State-Trait Anxiety Inventory (STAI), 12-item General Health Questionnaire (GHQ), Functional Assessment of Cancer Therapy-Head and Neck Scale (FACT H&N), and the visual analog scale (VAS).

**Results:**

The examined groups were homogenous and carefully matched in terms of mental health (GHQ), the quality of life (FACT H&N), and the intensity of depression level (DASS). The IONM group showed a significantly lower level of “the state anxiety”(STAI) 1 day before the operation (*p* < 0.05), greater trust in the doctor (VAS) (*p* < 0.05), and greater confidence in the treatment method (VAS) as compared to the patients in the control group (*p* < 0.05), while no significant differences were found when the remaining items were compared.

**Conclusions:**

The planned use of IONM during thyroidectomy may reduce patient anxiety before surgery. However, further research in this area is necessary to confirm this preliminary finding in a larger population of patients.

## Introduction

Stress and accompanying emotions, such as anxiety and depression, may provoke an increase in pro-inflammatory cytokines, which may induce immune dysregulation and increase the variety of diseases [1–7]. A few mechanisms linking stress to health outcomes have been identified. Two interacting routes are taken to account. The first route is direct, by activating the hypothalamic-pituitary-adrenal and the sympathetic-adrenal medullary axes, thereby resulting in downstream hormonal and immune changes. The second path is indirect, by inducing negative emotions, which in turn affects physiological processes [1].

Furthermore, a surgical injury produces a multidirectional stress response (the general adaptation syndrome—GAS) [8, 9]. Linn et al. proved that higher preoperative stress was associated with a lower lymphocyte response and lower lymphocyte counts in the blood following the surgery [10, 11]. Broadbent et al. found that higher psychological stress before the surgery predicted a lower cellular wound repair processes in the early postoperative period. Patients who reported a higher perceived stress 1 month before the surgery had lower levels of interleukin-1 in their surgical wounds [10]. Therefore, preoperative stress reduction due to the safer surgical method may be beneficial for the patients.

The use of intraoperative neuromonitoring (IONM) of the laryngeal nerves during thyroidectomy has the potential to improve patient safety. Attention is focused on minimizing the risk of intraoperative laryngeal nerve injury in thyroid surgery using IONM [12]. However, a comprehensive psychological comparison of preoperative stress in patients scheduled for thyroidectomy with versus without IONM has never been performed. The aim of this study was to find out whether the planned use of IONM affected reduction of the stress and anxiety level before and after thyroid surgery.

## Material and methods

### Study design and patients

This is a pilot study, which aims at exploring a new hypothesis. The demographic characteristics of patients involved in this study are presented in Table 1. Thirty-two patients scheduled for thyroidectomy with IONM (mean age 49.0 ± 15.3 years, education 13.0 ± 2.5 years on average, mean BMI 27.0 ± 5.3 kg/m^2^) were evaluated prior to the surgery with a set of standardized questionnaires. The results were compared to the outcomes of a carefully matched control group: 39 patents operated on without IONM (mean age 50.0 ± 12.3, education 13.0 ± 2.6 years on average, mean BMI 27.0 ± 4.7 kg/m^2^). Both groups were comparable with respect to gender, age, education, BMI and indications for surgery, and the extent of planned thyroid resection (total thyroidectomy). Both groups consisted of patients with benign nodular goiter, in euthyroid state, aged 25–65 years. All the patients included in the study received the same information on the surgery. The benefits and the risks of the proposed treatment were presented to the patients before the questionnaires. All the patients were tested 1 day before the operation and at 1–7 days following the surgery.

**Table 1:** Characteristics of patients

Groups	No.	Mean age ± SD (years)	F/M ratio	Education background ± SD (years)	BMI ± SD (kg/m^2^)
IONM (+)	32	49 ± 15.3	30:2	13 ± 2.5	27 ± 5.3
IONM (−)	39	50 ± 12.3	36:3	13 ± 2.6	27 ± 4.7

*IONM* intraoperative neuromonitoring, (+)—with, (−)—without

Patient selection was based on individual surgeon’s decision taking into consideration the risk of recurrent laryngeal nerve (RLN) injury. High-risk patients (revision thyroidectomy, substernal goiter, toxic multinodular goiter) were proposed the procedure with IONM, whereas low-risk patients (small or medium-sized non-toxic nodular goiter) were operated on without IONM.

This research was a multicenter study carried out in three participating centers. All thyroidectomies were performed by four surgeons with more than 10 years of experience in thyroid surgery, and well trained in using IONM. Psychological testing was performed by a single clinical psychologist (D.B.). The study protocol was approved by the Institutional Review Board. All the patients signed the informed consent prior to enrollment. The surgery and hospital stay were free for the patients, regardless of the method of operation.

### Psychological testing

The following psychological self-report instruments were used:

- Depression Anxiety Stress Scales (DASS) is a clinical psychology test, designed to measure the three related negative emotional states of depression (D-DASS), anxiety (A-DASS), and stress (S-DASS) (a coefficient of reliability, the so-called Cronbach’s alpha = 0.96, 0.89, and 0.93 for depression, anxiety, and stress, respectively) [13, 14].
- State-Trait Anxiety Inventory (STAI) measures anxiety. It clearly differentiates between the temporary condition of “state-anxiety” (A-State) and the more general and longitudinal quality of “trait-anxiety” (A-Trait) [15]. A-State is the changeable feeling adjusted for example to a dangerous situation. It can be defined as fear, nervousness, discomfort, and the arousal of the autonomic nervous system temporarily induced by situations perceived as dangerous (i.e., how a person is feeling at the time of a perceived threat) [16]. A-Trait is a certain general model of anxiety level as a stable personality trait. It may be defined as a relatively enduring disposition to feel stress, worry, and discomfort (Cronbach’s coefficient alpha = 0.84–0.87) [16].
- General Health Questionnaire (GHQ) (12-items) is a well-validated short version used to detect the degree of emotional distress. It is intended to screen general psychiatric morbidity; (coefficient alpha = 0.82–0.90 [17–20], and in the Polish adaptation—Cronbach’s coefficient alpha = 0.79–0.89) [17–20].
- Functional Assessment of Cancer Therapy-Head and Neck Scale (FACT H&N) is a multidimensional, self-report quality of life instrument; it assesses patient’s functioning in four domains: physical, social/family, emotional, and functional well-being, which is further supplemented by 12 site-specific items to assess head and neck-related symptoms (coefficient alpha = 0.63–0.89) [21–24].
- Visual analog scale (VAS) measures the subjective evaluation of the patient’s pain, mood, stress, fear and satisfaction from the treatment [25].

All of the abovementioned tests were used before the surgery. STAI and expanded VAS were the only tests used after the surgery.

### Statistical analysis

Data were presented as means and standard deviations, unless indicated otherwise. All continuous variables were evaluated with the Students *t* test. All other comparisons (nominal variables) were performed with the *χ* ^2^ test. Correlations were evaluated with the Spearman method. Evaluations were considered significant at *p* < 0.05. The statistical analysis was performed using the statistical software STATISTICA 10, by StatSoft Poland.

## Results

The comparison of results achieved employing psychological self-report instruments is shown in Table 2.

**Table 2:** Psychological self-report instruments results

	D-DASS (depression scale)	A-DASS (anxiety scale)	S-DASS (stress scale)	GHQ (sten scores)	FACIT N&H total results	A-State (STAI)	A-Trait (STAI)
IONM (+)	4.1	6.18	10.11	4	118.46	5.89	3.7
IONM (−)	4.0	5.63	9.76	4	109.19	6.72	4.0
*p* value	ns	ns	ns	ns	ns	0.05	ns
Norm	0–9	0–7	0–14	0–4	–	–	–

*ns* not significant

The majority of the psychological self-reports included in the study presented no significant differences before the surgery between the groups. All the patients were tested with DASS and GHQ in order to exclude the influence of depression on the questionnaire outcomes. There was no significant difference in the intensity of depression level between the study and control groups as presented by DASS. Both groups were within the clinical borders of “no depression” interpretation of DASS. There was no difference between the groups in GHQ results. There also was no significant difference in the level of life quality between the study and control groups, as presented by FACT-H&N total score. This may indicate that the examined groups were homogenous and carefully chosen in terms of mental health and the quality of life.

In the analyzed material, there was no significant difference in the intensity of A-Trait, S-DASS, and A-DASS, between the groups. It can mean that there was no important difference between the groups in the level of stress as the general model of anxiety level, (DASS and STAI). However, there was a significant difference in the intensity of A-State level between the groups before the operation (STAI). The study group had significantly lower results in A-State (*p* < 0,05), which may mean that patients in the IONM group showed a lower anxiety level, measured 1 day before the operation, as compared to the patients in the control group.

There was a crucial difference between the groups in the results of seven VAS scales measured before the operation (Table 3). It showed a higher level in the control group referring to “the fear of the treatment results”, “the fear of the voice loss”, “the fear of the size of the scar” “sadness”, “lower mood” “nervousness” compared to the IONM group. There were no important differences between the groups in such scales as “family support” and “motivation to the treatment.” The IONM group showed significantly higher results than the controls in “the trust in the doctor” and “the treatment method came up to their expectations”. There was no important difference between the groups after the operation in all these scales.

**Table 3:** The results of VAS preoperatively

VAS	IONM(+)	IONM(−)	*p*
“motivation to the treatment”	79.07	84.62	0.346
“lower mood”	13.57	27.15	0.022
“fear of the treatment results”	29.36	47.05	0.016
“fear of the voice loss”	41.84	63.27	0.006
“family support”	88.44	86.01	0.663
“nervousness”	17.14	32.17	0.035
“fear of the size of the scar”	20.66	43.33	0.004
“trust in the doctor”	92.41	80.71	0.007
“the treatment method came up to their expectations”	81.95	68.88	0.047

After the operation, both groups responded to the additional VAS about “the recovery time,” “the acceptance of the size of the scar”, “the ease of speaking” and “the ease of swallowing,” and “the general satisfaction with the operation”. There was no significant difference in these scales between the groups. It may suggest that both groups achieved similar satisfaction with the operation the patients had undergone.

**Fig. 1:**
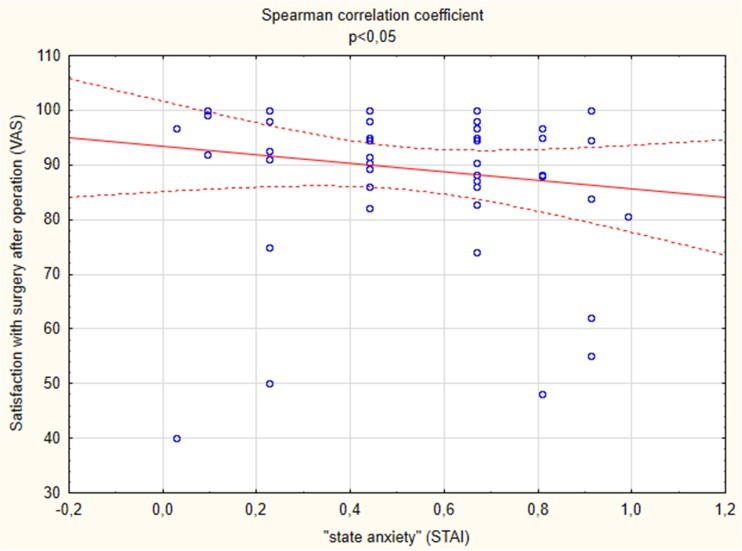
Significant negative correlation between A-state before the operation and “satisfaction with surgery” after the operation, as presented by VAS (*r* = −0.31324, *p* < 0.05)

The Spearman correlation coefficient indicated a significant negative correlation between A-State before the operation and “the satisfaction with the surgery” after the operation, as presented by VAS (*r* = −0.31324). The lower the anxiety before the surgery (evaluated on the day before the surgery), the higher the results in terms of overall satisfaction with the surgery, tested 1–7 days after the operation (as shown in Fig. 1).

This study also showed important correlations between A-State before the operation and the following data: “the fear of the size of scar” (*r* = 0.3298) measured before the surgery and “the easiness of speaking” measured after the surgery (*r* = −0.35137), as presented by VAS. It may suggest that the lower anxiety induced temporarily by situations perceived as dangerous, the lower “the fear of the size of scar” before the operation and the higher “the easiness of speaking” measured after the surgery.

The level of education was negatively correlated with D-DASS before the operation (*r* = −0.3651) in all the studied populations. It may suggest more years of education, a lower depression level before the surgery as presented by DASS. Neither age, nor education, significantly correlated with “the fear of the voice loss” as presented by VAS before or after the surgery.

In the analyzed material, we also found significant negative correlations between the quality of life as presented by FACT-H&N total score, and A-Stait (*r* = −0.3804), A-Ttrait (*r* = −0.5895), D-DASS (*r* = −0.7183), A-DASS (*r* = −0.6343), S-DASS (*r* = −0.5482), “nervousness” (*r* = −0.4117), and “the fear of the treatment results” (*r* = −0.278), as presented by VAS. It may suggest that the higher level of life quality, the lower anxiety (as a general model of anxiety and as a temporary condition just before the surgery), the lower depression level, “nervousness” and “the fear of the treatment results,” before the surgery.

None of the patients in this study had any postoperative complications.

## Discussion

To the best of our knowledge, this is the first report in the literature which proved that the planned use of IONM during thyroidectomy may reduce patients’ anxiety before surgery. An adequate and well-controlled psychological study has never been undertaken in this field. The results indicate that the examined groups were homogeneous and carefully chosen in terms of mental health and the quality of life. These factors probably did not affect or disturb the self-assessment of the stress and anxiety level caused by the waiting for the operation.

The present study indicated that the IONM group showed a lower anxiety level (as temporary condition just before the surgery), higher “satisfaction from the undergone operation”, and “the easiness of speaking” after the surgery than patients in the control group. The IONM patients also demonstrated a higher the level of general life quality, lower stress and depression levels, a lower general model of anxiety, and a temporary condition of anxiety, caused by waiting for the surgery.

However, differences between some results could be confusing, as far as the anxiety results before the surgery are concerned. There were significant differences between the groups in A-Stait results, but there were no crucial differences in A-DASS and A-Trait results. The reason for the differences in these results is as follows: Depression Anxiety Stress Scale evaluates the anxiety (A-DASS) from longer interval—“last 7 days,” similarly as Stait-Trait Anxiety Inventory evaluates the A-Trait level. Only A-Stait evaluates differently—it assesses anxiety in a shorter interval—“just today” (patient’s condition 1 day before the surgery). It may suggest that the groups are also homogenous in terms of relatively enduring disposition to experience stress, worry, and discomfort in the period of 7 days before hospitalization.

A-State is well-known to be a sensitive predictor of distress over time; it may vary in health changes [26, 27]. Therefore, the patient’s psychological state before the operation may have an important influence on the treatment results and recovery.

The majority of the psychological instruments used in this study are standardized and have proper reliability. Cronbach’s alpha is the most common measure of internal consistency (“reliability”) of a psychological test or questionnaire. The chosen psychological instruments show the values of Cronbach’s alpha above 0.7 (the maximum possible value is 1). This confirms the reliability of the test (all items are reliable and measure the expected issues). Some people think that the research setting and the way of administering the VAS forms may not be convincing [28, 29]. However, the VAS method is used in the clinical, social, and behavioral sciences to measure a variety of subjective phenomena or attitudes that cannot be directly measured. The conceptual, psychometric, and statistical aspects of the VAS are considered [25]. Clinician-rated measurements of mood are an accepted standard; self-report of symptoms provide complementary and important information on the course and variability of illness from the patient’s perspective [30].

The major limitation of this study is that the patients were not randomly assigned to undergo the surgery with vs. without IONM. Randomization was not performed due to ethical reasons, as it would not be reasonable to include high-risk patients into the arm of the study operated on without IONM. Instead, patient selection was based on individual surgeon’s decision taking into consideration the risk of RLN injury. High-risk patients were offered thyroid surgery with IONM, whereas low-risk patients were operated on without IONM. This might have led to a selection bias. However, the outcomes of this study showed a lower level of preoperative anxiety in high-risk patients operated on with IONM (who in theory should experience higher preoperative stress) when compared to low-risk patients operated on without IONM (who in theory should experience lower preoperative stress). Hence, in high-risk individuals, the patients’ awareness of complexity of their case must have been balanced by the information about the planned utilization of IONM during the surgery, which lowered the anxiety level. Therefore, the outcomes of this pilot study may be interpreted as supporting the hypothesis explored in this research.

The limitation of our study may be associated with the fact of conveying information to the patient before the surgery. The study was conducted at three medical centers and by four experienced surgeons. Despite creating standard information about the two methods of operation, the advantages and disadvantages of each method, information could have been presented a little differently in each center. The reason may be different personalities of doctors, their different ways and skills of communication. The reason may also be different intellectual potential of the patients, their skills to cope with preoperative stress. A high level of stress may impair the functioning of cognitive processes, and change the evaluation of risks and receiving information [31, 32]. A higher overall level of preoperative stress may influence the evaluation of “the fear of the size of the scar” in patients operated without IONM; although rationally, we can see that the size of the scar does not depend on the use of IONM.

The operations were conducted by four different surgeons. Maybe it would be useful to analyze the data reported to find the statistical difference between surgeon groups in all the scales. It would not be reliable in such a small group. However, it is worth remembering that the surgeons are experienced specialists, who work according to required procedures. The presence of several surgeons (from various centers) makes the results more versatile.

The limitation of our study is also visible in the postoperative examination. The patients were assessed 1–7 days after the surgery. Perhaps the same patient would have provided different answers a day and 7 days after the surgery, e.g., in the field of pain. However, the results of our study suggest no differences in the patient assessment of “the recovery time,” “the acceptance of the size of the scar”, “the ease of speaking” and “the ease of swallowing,” and “the general satisfaction with the operation” at various times after surgery (from 1–7 postoperative days). This may suggest that the patients agree with this opinion, regardless the time they were questioned after the operation.

## Conclusions

The results of this pilot study suggest that the planned use of IONM during thyroidectomy may reduce patients’ anxiety before the surgery. The lower the patient’s anxiety level (as a temporary condition just before the surgery), the higher “the satisfaction from the undergone operation”, and “the easiness of speaking” measured after the surgery. In addition, the higher the level of general life quality, the lower the stress and depression level, the lower the general model of anxiety and a temporary condition of anxiety, caused by waiting for the surgery. However, further research in this area is necessary to confirm this preliminary finding in a larger population of patients.

## Abbreviations

IONM: Intraoperative neuromonitoring
DASS: Depression Anxiety Stress Scales
GHQ: General Health Questionnaire
VAS: Visual analog scale
STAI: State-Trait Anxiety Inventory
FACT H&N: Functional Assessment of Cancer Therapy-Head and Neck Scale
BMI: Body mass index
GAS: The general adaptation syndrome
